# Introducing Community Houses in Italy: a scoping review of the international literature

**DOI:** 10.1093/eurpub/ckac130.011

**Published:** 2022-10-25

**Authors:** A Cerri, P Arcaro, E Veneziano, A Panà, R Mete, M Maurici, C De Vito, G Damiani

**Affiliations:** 1 Department of Public Health, Sapienza University of Rome, Rome, Italy; 2 Department of Life Sciences and Public Health, Catholic University of the Sacred Heart, Rome, Italy; 3 Department of Biomedicine and Prevention, University of Rome Tor Vergata, Rome, Italy; 4 Departmental Faculty of Medicine and Surgery, Campus Bio-Medico University of Rome, Rome, Italy; 5 Istituto Superiore di Studi Sanitari Giuseppe Cannarella, Rome, Italy

## Abstract

**Background:**

The SARS-CoV-2 pandemic put under pressure all the world's health systems, to the point that it was a severe threat to their stability. At the same time, this scenario confirmed the importance of primary health care to guarantee effective care for patients who suffer from complex and chronic diseases. From these considerations and in the light of the funding provided by the European Union for enhancing the health care system in Italy, our working group has decided to analyse various organisational models of Primary Health Care founded around the world to set up innovative Primary care community Centers in Italy, called Community Houses.

**Methods:**

A scoping review of the international literature was conducted on Pubmed, searching for primary care models based on integration and co-location of services. Each organisational model was then evaluated using different levels of multidimensional integration inspired by the taxonomy work of P. P. Valentijn, such as clinical, professional, organisational, system, functional and normative integration levels.

**Results:**

The search produced 2053 results, initially screened by title and abstract and, subsequently, by full-text, finally obtaining 116 articles. When a model is characterised by integrating services with external stakeholders, it also presents more integration levels than the others. In particular, these models are, on average, about 20% more likely to have an organisational, functional and normative integration in the model. Moreover, by stratifying for population complexity, we can find an increase in integration levels for populations suffering from chronic diseases with a higher degree of complexity, such as diabetes or cancer.

**Conclusions:**

From these preliminary results, we can conclude that it is necessary to prefer primary care models with more integration levels to deliver better healthcare for people with complex or chronic diseases, improving the performance of the Health Care System, especially in Italy.

**Key messages:**

- Our work has allowed us to advise the policymakers on a correct reorganisation of the National Health System in Italy employing the European Union funds.
- The results of this study will be used to organised Primary care community Centers in Italy, called Community Houses, which aim to facilitate the integration and co-location of services.

